# Estimating the asphaltene critical nanoaggregation concentration region using ultrasonic measurements and Bayesian inference

**DOI:** 10.1038/s41598-021-85926-8

**Published:** 2021-03-23

**Authors:** Aleksandra Svalova, David Walshaw, Clement Lee, Vasily Demyanov, Nicholas G. Parker, Megan J. Povey, Geoffrey D. Abbott

**Affiliations:** 1 School of Mathematics, Statistics and Physics, Newcastle University, Newcastle upon Tyne, NE1 7RU UK; 2 School of Natural and Environmental Sciences, Newcastle University, Newcastle upon Tyne, NE1 7RU UK; 3 Institute for GeoEnergy Engineering, Heriot-Watt University, Edinburgh, EH14 4AS UK; 4 Department of Mathematics and Statistics, Lancaster University, Lancaster, LA1 4YF UK; 5 School of Food Science and Nutrition, University of Leeds, Leeds, LS2 9JT UK

**Keywords:** Environmental impact, Fossil fuels, Statistics

## Abstract

Bayesian inference and ultrasonic velocity have been used to estimate the self-association concentration of the asphaltenes in toluene using a changepoint regression model. The estimated values agree with the literature information and indicate that a lower abundance of the longer side-chains can cause an earlier onset of asphaltene self-association. Asphaltenes constitute the heaviest and most complicated fraction of crude petroleum and include a surface-active sub-fraction. When present above a critical concentration in pure solvent, asphaltene “monomers” self-associate and form nanoaggregates. Asphaltene nanoaggregates are thought to play a significant role during the remediation of petroleum spills and seeps. When mixed with water, petroleum becomes expensive to remove from the water column by conventional methods. The main reason of this difficulty is the presence of highly surface-active asphaltenes in petroleum. The nanoaggregates are thought to surround the water droplets, making the water-in-oil emulsions extremely stable. Due to their molecular complexity, modelling the self-association of the asphaltenes can be a very computationally-intensive task and has mostly been approached by molecular dynamic simulations. Our approach allows the use of literature and experimental data to estimate the nanoaggregation and its credible intervals. It has a low computational cost and can also be used for other analytical/experimental methods probing a changepoint in the molecular association behaviour.

## Introduction

Petroleum spills occur due to anthropogenic (as well as natural) phenomena, such as petroleum exploration, transportation and refining^1–3^. The long-term impacts to the ecosystem by oil spills are reviewed elsewhere^4–6^. Water-in-oil emulsions (WOE) form during petroleum spills as a result of petroleum mixing with sea water, whereby very little energy is required for emulsification to occur^7^. Such emulsions are very stable and problematic to remove due to their high viscosity and stability^8, 9^. The efficient removal of WOEs thus requires phase separation into water and oil phases. The WOE stability is a function of factors that include a high water content (30–90%)^10, 11^, water salinity^12, 13^ and pH^14, 15^.

Asphaltenes, and specifically the natural interfacially-active emulsifiers within them^16, 17^, have been extensively reported to be the main cause of the high WOE stability. The importance of waxes and the water droplet size distribution in increasing the viscosity of WOEs has also been reported numerously^11, 18^. Asphaltenes is a class of compounds that is operationally defined as soluble in toluene and insoluble in *n*-pentane or *n*-heptane^19–21^. The features of an asphaltene fraction, therefore, are defined by the precipitating solvent and can comprise a vast structural polydispersity^19, 21, 22^. A subfraction of the asphaltenes^23, 24^, that is reported to be more polar^25^, stabilises the WOEs by adsorbing at the water/oil interface forming rigid films resisting droplet coalescence^12, 26, 27^.

In understanding the asphaltene phase properties, the Yen-Mullins model^20^ is one of the most widely-accepted. It suggests that in low concentrations, asphaltenes in petroleum exist as monomers. As their concentration increases to the critical nanoaggregate concentration (CNAC) of 50–150 mg/L asphaltenes self-associate into nanoaggregates. Further, as their concentration reaches 2–3 g/L asphaltenes start forming clusters^20^. The surface-active asphaltenes self-associate at the nanoscale^28^ forming nanoaggregates, the latter were reported to form films that stabilise WOEs^29–31^. The nanoaggregates are ca. 3–10 nm in size, have an ellipsoidal shape and can entrap solvent within the aggregate interior^32, 33^. There are two primary forces governing nanoaggregation, attraction between the aromatic cores and repulsion from the aliphatic appendages^20^. We have previously suggested that the abundance of longer side-chains ($\hbox {C}_{\ge 19}$) contributes to a later onset of nanoaggregation^34^. Asphaltene nanoaggregates have been suggested to contribute to the stability of water-in-oil emulsions^20, 35, 36^. The asphaltenes’ nanoaggregate state at the water-oil interface has also been debated, proposing that molecules are in a monomeric^37, 38^ state or the observed film density is grater than that of the nanoaggregate (although the nanoaggregate thickness is preserved)^16^. A discussion about this and further literature review of asphaltene nanoaggregation is presented in Svalova et al.^34^. In what follows it is assumed that asphaltene nanoaggregates contribute to the WOE stability.

Asphaltene nanoaggregation is assumed to be the first stage of their self-association, which occurs at concentrations of ca. 100 mg/L ($\pm 50\ \hbox {mg}/\hbox {L}$) in toluene^20, 39, 40^, corresponding to the critical nanoaggregate concentration (CNAC)^39^. This concentration can be determined using conductivity^41^, centrifugation^42^, nuclear magnetic resonance^43^ and high-*Q* ultrasonic measurements^39^. We have used the latter technique to test the CNAC of four asphaltene samples^34^. At higher concentrations (g/L) asphaltene nanoaggregates start forming clusters corresponding to the critical cluster concentration.

The question of asphaltene self-association and aggregation has been approached by modelling methods mainly using molecular dynamics. Jiménez-Serratos et al.^44^ used coarse-grained molecular simulations and the statistical associating fluid theory equation of state^45^ to investigate the impact of asphaltene concentration, solvent composition and temperature on aggregation. Coarse-grained molecular simulations^46^ indicated an agreement with the Yen–Mullins hierarchy^20^ detecting nanoaggregation and clustering, whereby asphaltene nanoaggregates with long aliphatic appendages could not form clusters. The characterisation of different stages of asphaltene aggregation in heptane were studied by umbrella sampling of the potential mean force^47^. The results suggested that in small-scale systems the formation of nanoaggregates occurs in less than 10 ns, with 4–12 monomers per nanoaggregate. Nanoaggregate formation was also observed in a large-scale system^47^. In contrast, Headen et al.^48^ used molecular dynamics to suggest that the distribution of asphaltene aggregates is continuous in character.

To our knowledge, however, there has been little effort yet to address the uncertainty associated with asphaltene nanoaggregation concentration in a probabilistic manner. As experiments are often costly and/or time-consuming, replication is not possible which makes statistical inference difficult. The latter is, however, possible when a Bayesian approach is deployed which makes use of expert/literature information as well as experimental data. This study focuses on a novel application of Bayesian inference better estimating the CNAC nanoaggregation concentration and its credible region using ultrasonic characterisation^39^. Ultrasonic chracterisation data of four asphaltene samples E1–E4^34^ will be used in this work.

In what follows we model the asphaltene nanoaggregation concentration assuming a piecewise regression with a single changepoint. The latter is equivalent to the model by Zielinski et al.^49^ used in earlier studies^34, 39^ and the changepoint (further denoted $\gamma$) is equivalent to the CNAC. Bayesian inference will be used to estimate the $\gamma$ mean and range as well as other model parameters using Markov chain Monte Carlo (MCMC) sampling. To avoid confusion, the asphaltene samples E1–E4 will be referred to as **specimens** and numerical values drawn by the MCMC algorithm will be referred to as **samples**. The Methods section describes our proposed model and the details of the sampling scheme. The section also refers to a synthetic study that we performed to verify the reliability of our sampler. The Results section illustrates illustrate the MCMC sampling outputs, including estimation of the posterior distribution of $\gamma$. The Discussion section further explores the results and compares them with the structural properties of the asphaltenes^34^. The combination of the asphaltene structural properties and a probabilistic estimation of nanoaggregation could be very useful in petroleum spill remediation strategies. Finally, in the Conclusions section we summarise the study and outline further directions.

## Methods

### Specimen information and preparation

**Figure 1:**
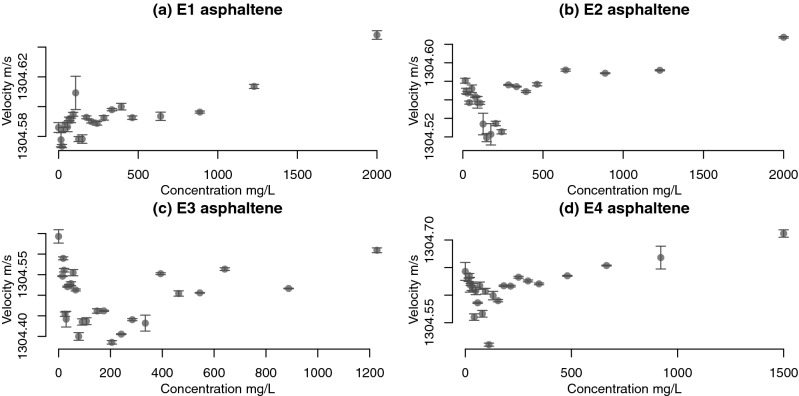
Ultrasonic velocity characterisation of asphaltenes E1–E4^34^, bars indicate 95% confidence intervals. In plot (**c**), one observation at c.a. 150 mg/L that was previously reported in^34^ was censored as it was an outlier in comparison to the remaining data.

The asphaltenes were precipitated from four petroleum samples: E1 with E2 and E3 with E4 are from two different source rocks respectively and all are from different reservoirs. E1 and E2 are from South America and E3 with E4 are from North America. The asphaltene preparation procedures, including precipitation and purification, geochemical description of the samples and ultrasonic methodology can be found in our earlier study^34^. The Resoscan^50^ ultrasonic instrument was used for the asphaltenes’ characterisation, the measurements are illustrated in Fig. 1^34^. Parent petroleum specimens were selected such that there are two specimens per source rock of difference degree of biodegradation. The specimens E1 (mildly biodegraded) with E2 (mildly-moderately biodegraded) and from E3 (highly biodegraded) with E4 (mildly-moderately biodegraded) are from two different source rocks respectively and all are from different reservoirs^34^. Noteworthy, there was significant noise in the data which we removed before modelling the data by analysing the outliers in the ultrasonic trace versus time. However diversions from the two-line model remain e.g. in the high-concentration tail of E3. These diversions may have been caused by the high molecular heterogeneity of the asphaltenes^51^, micro air bubbles^52^ or trace impurities. This is illustrated numerically in our synthetic data study where we emulate one of our specimens and a specimen from Andreatta et al.^39^ to validate our MCMC scheme. Bayesian inference can serve to remediate this as it combines expert opinion^40^ and experimental data to estimate the aggregation point. The synthetic data study also illustrates this as the aggregation point is recovered for both data sets.

### Bayesian inference using Markov chain Monte Carlo sampling

This subsection provides an introduction to Bayesian inference and Markov chain Monte Carlo simulation which the familiar reader should feel free to skip.

In statistical modelling, we assume a process/experiment *Y* generating an outcome *y* that is governed by a model *M* and controlled by a collection of parameters (vector) $\varvec{\theta }$. Statistical inference is carried out to infer/estimate $\varvec{\theta }$, of which there are two main approaches: frequentist and Bayesian. The frequentist/classical approach is based on how the process would behave given a high number of repetitions *n*, and has been used in our previous analysis^34^. Often, however, experiments and/or events cannot be repeated (a meaningful number of times). The Bayesian approach^53^ is extremely useful in conditions of low/noisy data availability and will be used here.

Required in both approaches is the likelihood. To formulate the likelihood, it is assumed that *Y* follows some probability distribution (e.g. Gaussian) with a probability density function *f*, and the probability of $Y=y$ under the model *M* controlled by $\varvec{\theta }$ is expressed by $f(y|\varvec{\theta })$ (*M* suppressed in notation). The likelihood function of $\varvec{\theta }$ given data $y=(y_1,y_2,\ldots ,y_n)$ is defined as the product of *f* over all data values of *y*, $L(\varvec{\theta }|y)=\prod _{i=1}^nf(y_i|\varvec{\theta })$.

In the likelihood approach, *L* is maximised to obtain the *maximum likelihood estimate* (MLE) of $\varvec{\theta }$ assuming *M*. This implies that the likelihood alone can be used to draw inference about $\varvec{\theta }$ and whether *M* is suitable to model *y*. However, when *n* is small then the uncertainty around the MLE will be high. The Bayesian approach, on the other hand, allows the incorporation of expert/literature knowledge about $\varvec{\theta }$, expressed in a probability distribution $\pi (\varvec{\theta })$, called the prior belief/distribution. The goal of inference is then to obtain the conditional probability distribution of $\varvec{\theta }$ given *y*. This distribution is also known as the *posterior distribution*, which is denoted by $\pi (\varvec{\theta }|y)$ and obtained through the Bayes’ rule^53^:

1 $$\begin{aligned} \pi (\varvec{\theta }|y)=\frac{\pi (\varvec{\theta }) L(\varvec{\theta }|y)}{\int \pi (\varvec{\theta }) L(\varvec{\theta }|y)dy}\propto \pi (\varvec{\theta })L(\varvec{\theta }|y). \end{aligned}$$

Formalism (1) can also be interpreted as the combination of the likelihood $L(\varvec{\theta }|y)$ and the expert knowledge $\pi (\varvec{\theta })$. Except for the simplest statistical models, the integral $\int \pi (\varvec{\theta } L(\varvec{\theta }|y)dy$ is usually analytically unavailable, thus making $\pi (\varvec{\theta }|y)$ analytically unavailable too. Therefore, Bayesian inference usually resorts to computational methods, among which Markov chain Monte Carlo (MCMC)^54^ is the most popular.

**Figure 2:**
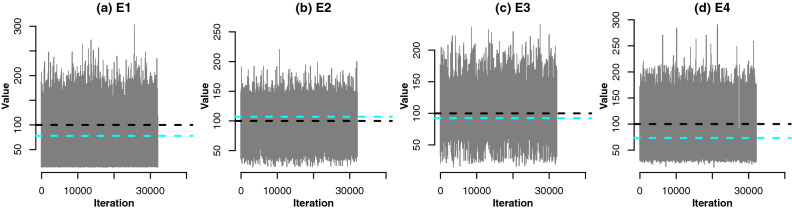
Trace plots of the posterior distribution of the changepoint $\gamma$ for E1–E4. Black lines indicate prior means, blue lines indicate posterior means.

The principle of MCMC is to provide random samples which *represent/approximate* the posterior distribution $\pi (\varvec{\theta }|y)$, through an iterative algorithm^54^. Specifically, the random samples are generated according to a Markov chain, which is a stochastic process whereby the value (of $\varvec{\theta })$ at state *i* only depends on the value at state $i-1$. The burn-in^54^ is the initial period of the chain whereby extreme/implausible values are likely to be accepted, thus the burn-in is typically discarded from the posterior analysis. The algorithm should be run long enough to achieve convergence, i.e. the distribution $\pi (\varvec{\theta }|y)$ approximated by the samples does not change with the new samples^54^. To find a reliable posterior estimate of $\varvec{\theta }$ it is essential that the Markov chain explores the space of $\varvec{\theta }$ efficiently. Posterior trace plots e.g. Fig. 2, where the samples are well-spread and appear uncorrelated with past values are an indication of high sampling efficiency. The effective sample size (ESS)^55^, e.g. Table 2, can also be used as a diagnostic whereby an ESS that is as high as the number of iterations imply maximum efficiency and an absence of autocorrelation between the samples.

**Figure 3:**
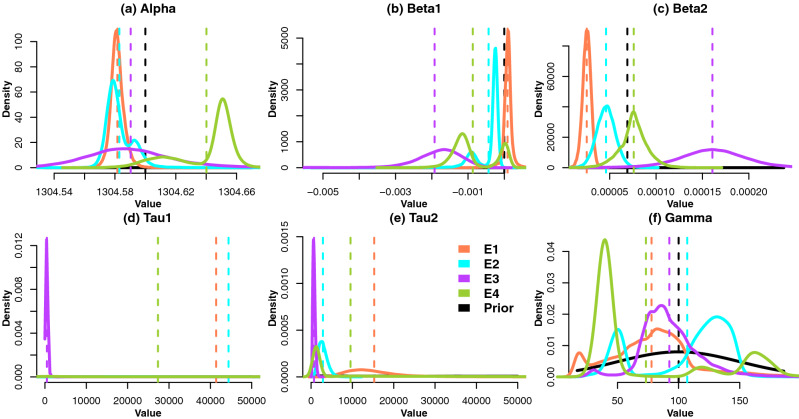
Density plots of the posterior distributions of the single changepoint model parameters. Dashed lines indicate means.

**Figure 4:**
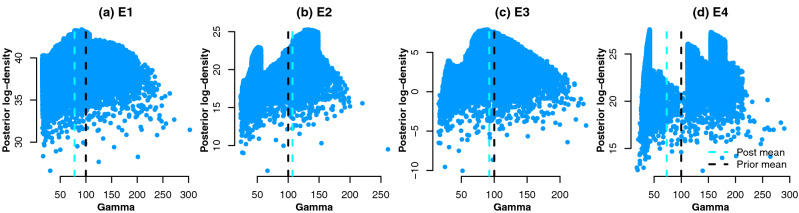
Plots of the posterior log-density against sampled values of $\gamma$ for E1–E4.

The density plots e.g. Fig. 3 and scatter plots e.g. Fig. 4 of the sampled values illustrate the posterior distribution $\pi (\varvec{\theta }|y)$, as well as how it differs to the prior beliefs. Using the MCMC samples that represent $\pi (\varvec{\theta }|y)$, we can obtain the posterior predictive distribution^56^ of a quantity of interest $\tilde{y}$:

2 $$\begin{aligned} \pi (\tilde{y}|\varvec{\theta })=\int \pi (\varvec{\theta }|y)L(\varvec{\theta }|\tilde{y})d\varvec{\theta }. \end{aligned}$$

**Figure 5:**
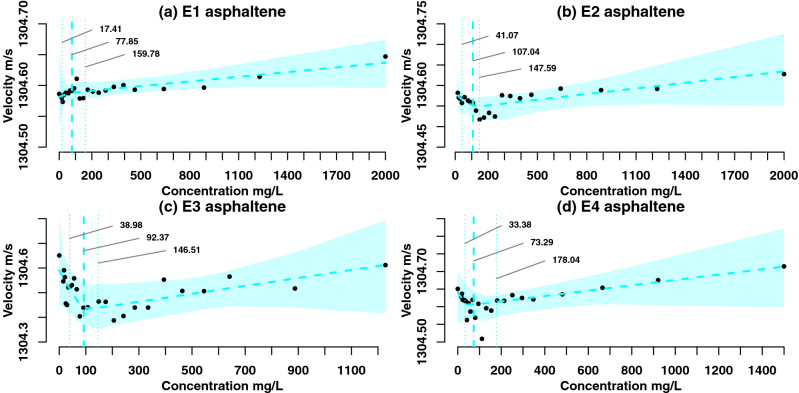
Posterior fit of the single changepoint model. Cyan shading indicates the Bayesian credible intervals, dashed line indicates mean model fit. Vertical cyan dashed lines indicate the posterior mean and 95% confidence interval of the changepoint $\gamma$.

This is how the posterior predictive intervals in Fig. 5 are obtained.

In the next section, we will specify $\varvec{\theta }$ and *y* in the context of a single changepoint model for the nanoaggregation of the asphaltenes. In the Prior elicitation and posterior inference section, we will elicit the prior distributions $\pi (\varvec{\theta })$ and specify the details of MCMC, when inferring the parameters of the changepoint model using the Bayesian approach.

### Single changepoint model

Nanoaggregation of the asphaltenes may be detected by ultrasonic velocity measurements using theory of surface-active compound (surfactant) aggregation^39, 49^. Within a uniform liquid, the ultrasonic velocity *u* is related to density $\rho$ and adiabatic compressibility $\beta$ of the medium according to the Urick equation^57^

3 $$\begin{aligned} u=\sqrt{\frac{1}{\rho \beta }}. \end{aligned}$$

For multi-phase fluids which are well-dispersed, and ignoring the effects of sound scattering (valid for sufficiently low concentration of scatterers and away from scattering resonances)^52^, Equation (3) can be applied with density and compressibility represented by weighted averages of the mixture components. An extension of Equation (3) allows to detect the onset of surfactant aggregation into micelles to detect the critical micelle concentration, as proposed by Zielinski et al.^49^, where the full model derivation is given. Without loss of generality, we present the Zielinski et al.^49^ model in the context of the asphaltene critical nanoaggregation concentration (CNAC) only. In particular, the sound velocity *u* is related to apparent molar solution quantities following the relation

4 $$\begin{aligned} u=u_0+\frac{u_0}{2}\left( \tilde{v}_1\left( 2-\frac{\tilde{\beta }_1}{\beta _0}\right) -v_0\right) c_1 +\frac{u_0}{2}\left( \tilde{v}_a\left( 2-\frac{\tilde{\beta }_a}{\beta _0}\right) -v_0\right) c_a, \end{aligned}$$

where *v* denotes specific volume, *c*- weight concentration, tilde- apparent quantities and subscripts refer to solvent (0), monomer (1) and aggregated (a) quantities. Also,

5 $$\begin{aligned} {\left\{ \begin{array}{ll} \text {if}\,\, c\le \text {CNAC},\,\,\text {then}\,\, c_1=c, \,\,\text {and}\,\,c_a=0,\,\, \text {otherwise}\\ \text {if}\,\, c > \text {CNAC}, \,\,\text {then}\,\, c_1=\text {CNAC},\,\,\text {and} \,\, c_a=c-\text {CNAC}. \end{array}\right. } \end{aligned}$$

The model (4) implies that pre- and post-aggregation, sonic velocity is related to surfactant concentration as a combination of two linear behaviours whose intersection estimates the CNAC.

Formalism (4) can be estimated by a single-changepoint linear regression model, where the speed of sound *y* varies with asphaltene concentration *x* as follows.

M0 $$\begin{aligned} y_i=\left\{ \begin{array}{l} \alpha _1 + \beta _1 x_i + \varepsilon _{1,i}, \quad \varepsilon _{1,i}\sim N(0,\tau _1^{-1}),\quad x_i<\gamma ,\\ \alpha _2 + \beta _2 x_i + \varepsilon _{2,i}, \quad \varepsilon _{2,i}\sim N(0,\tau _2^{-1}),\quad x_i\ge \gamma ,\\ \end{array}\right. \end{aligned}$$

where $i=1,2,\ldots ,n$ denotes the sample index, *n* denotes the total number of measurements, $\varepsilon _{j,i},\, j=1,2$ refers to random errors that follow a Normal distribution with mean 0 and precision $\tau _j$, subscripts refer to the monomeric ($j=1$) and aggregated ($j=2$) concentrations respectively. Given that Model (M0) requires that the two regression lines intersect at $x=\gamma$, there is an identifiability issue with the quintuplet $\{\alpha _1,\alpha _2,\beta _1,\beta _2,\gamma \}$ as any of the parameters can be defined as a combination of the remaining four. We set $\alpha _2=\alpha + (\beta _1-\beta _2)\gamma$, $\alpha =\alpha _1$ and further use the formalism

M1 $$\begin{aligned} y_i=\left\{ \begin{array}{rl} &{}\alpha +\beta _1 x_i +\varepsilon _{1,i}, \quad \varepsilon _{1,i}\sim N(0,\tau _1^{-1}),\quad x_i<\gamma ,\\ &{}\alpha + (\beta _1-\beta _2)\gamma +\beta _2 x_i +\varepsilon _{2,i}, \quad \varepsilon _{2,i}\sim N(0,\tau _2^{-1}), \quad x_i\ge \gamma . \end{array}\right. \end{aligned}$$

In M1, $\gamma$ denotes the changepoint (equivalent to CNAC). Note that $\alpha$ corresponds to the speed of sound in pure solvent (toluene). Let $\theta =(\alpha ,\beta _1,\beta _2,\gamma , \sigma _1,\sigma _2)$ and the likelihood for M1 is as follows:

6 $$\begin{aligned} L(\theta |x,y)=\prod _{i=1}^{n_1}\sqrt{\frac{\tau _1}{2\pi }}\exp \left\{ -\frac{\tau _1(y_i-\alpha -\beta _1 x_i)^2}{2}\right\} \times \prod _{i=n_1+1}^{n}\sqrt{\frac{\tau _2}{2\pi }} \exp \left\{ -\frac{\tau _2\left( y_i-\alpha - (\beta _1-\beta _2) \gamma - \beta _2 x_i\right) ^2}{2} \right\} . \end{aligned}$$

In the above, $n_1$ denotes the size of $\{x \ni x_i <\gamma ,i=1,2,\ldots n_1 \}$ and *n* is the total number of measurements (sample size).

### Prior elicitation and posterior inference

The prior distributions $\pi (\theta )$ for the model parameters $\theta$ were elicited using the information in^39^ summarised in Table 1. This data can be used to define prior distribution means.

**Table 1:** Regression coefficients^39^ assuming a model by^49^.

Name	$\alpha$ (m/s)	$\beta _1$ (mg/L)	$\beta _2$ (mg/L)	$\gamma$ (mg/L)
UG8 asphaltene	1307.099	$-2\times 10^{-6}$	5.9$\times 10^{-5}$	164
BG5 asphaltene	1307.099	$-4\times 10^{-6}$	7.9 $\times 10^{-5}$	48
Tween 80	1307.121	NA	NA	NA
Brij 35	1307.446	NA	NA	NA

Tween 80 ($\hbox {C}_{30}\hbox {H}_{56}\hbox {O}_{9}$) and Brij 35 $(\hbox {C}{20}\hbox {H}_{42})_{5}$ are names for model surfactant compounds whose toluene solutions were also analysed using ultrasonic characterisation.

Noteworthy, the values of $\beta _1$ and $\beta _2$ are multiplied by a factor of $10^{-3}$ as our concentration values are in mg/L rather than in g/L as in^39^. Also note that $\alpha$ is equivalent to the speed of sound in pure toluene/solvent measured by a specific instrument. We suggest that if the information on the speed of sound in toluene is available for a given instrument then that information is used for the prior mean of $\alpha$ is used instead of that in Table 1.

Prior/expert distributions are chosen to allow conditional posterior distributions to be analytically available where possible:

7 $$\begin{aligned}&\alpha \sim N(a_1=1304.6,s^2_{\alpha _1}=10^2),\quad \beta _1\sim N(b_1=-3 \times 10^{-6},s^2_{\beta _1}=10^2),\quad \beta _2\sim N(b_2=6.9\times 10^{-5},s^2_{\beta _2}=10^2), \end{aligned}$$

8 $$\begin{aligned}&\tau _1\sim Ga(\rho _1=1,\phi _1=10^{-5}),\quad \tau _2\sim Ga(\rho _2=1, \phi _2=10^{-5}), \quad \gamma \sim N(g=100,s^2_{\gamma }=50^2), \end{aligned}$$

where *N* and *Ga* denote normal and Gamma distributions respectively. The prior mean and standard deviation of $\gamma$ (equivalent to the CNAC) are elicited from literature^20^. The mean of the $\alpha$ is set at the mean speed of sound in toluene by using our ultrasonic instrument^34^. All the parameters apart from $\gamma$ can be sampled from their conditional posterior distributions using a Gibbs step^54^, details of which can be found in the Supplementary Information (Equations (S1–S5)). The conditional posterior distribution of $\gamma$ is not analytically available thus will be sampled using a Metropolis^54^ update. The MCMC sampler was written in R statistical software^58^ and C++ through the Rcpp package^59^.

We carried out a study (Supplementary Information) on synthetic data designed to emulate the UG8 asphaltene by Andreatta et al.^39^ and our E2 specimen. Noteworthy, the precision value to emulate the E2 specimen was found to be two orders of magnitude lower than that of UG8, Figure S1. In other words, the synthetic data study illustrated that there is significant noise in our data compared to that of^39^. The synthetic study included testing the impact of prior mean misspecification on the posterior estimation of $\alpha$. We used the mean speed of sound in toluene for our samples (1304.6 m/s) as the prior mean, which is 2.5 m/s different do that in UG8. As asphaltene ultrasonic characterisation within a concentration range of 0–2000 mg/L corresponds to a velocity range size of less than 0.1 m/s, the said difference of 2.5 m/s is twenty-five times a typical velocity measurement range. Despite this, our sampling scheme correctly recovered the true value of $\alpha$ for both data sets.

The chain mixing was very efficient for all parameters (Figure S2), and all posterior densities illustrate a single mode (Figure S3). The only exception is the posterior distribution of the synthetic E2 for $\gamma$ which has a number of closely-distributed local peaks. This might be related to the compartmentalisation of the model posterior mode around specimen data in conditions of noise, Figure S4. As the amount of prior information for the regression coefficients is low, we chose very flat (high variance) prior distributions of monomeric and aggregated regression precisions. The synthetic study illustrated that this prior distribution allows to recover true precision values with a difference of two orders of magnitude. The posterior predictive regions for both specimens are illustrated in Figure S5 where the 95% regions reflect the magnitude of noise/uncertainty associated with each of the specimens.

## Results

A Metropolis-within-Gibbs MCMC scheme^54^ based on Equations (S1)–(S5) was used to sample the joint posterior distribution of the regression coefficients and the changepoint $\gamma$. The scheme was run for a burn-in period of $10^{6}$ iterations^54^, after which $4\times 10^{4}$ samples were obtained after thinning by 100. An exception is the E4 specimen which was thinned by 1000 due to extremely poor mixing and high autocorrelation. In particular, the effective sample size (ESS)^55, 60, 61^ of $\alpha$ was c.a. 150. In comparison, given totally uncorrelated sample the ESS should be very close to the MCMC chain length. Figure 2 illustrates the mixing for the changepoint $\gamma$ for all specimens. Good mixing can be observed in all cases and the sample space is sufficiently explored. Mixing plots for the remaining parameters can be found in Supplementary Information (Figure S6), where mixing is good in all cases except for $\tau _1$ of E3. In the latter, the sampler is exploring the extreme range of $\tau _1$ potentially caused by the high noise in the monomeric regression region. The ESS of the thinned MCMC runs is greater than 1300 for all parameters which is also satisfactory, as in Table 2. The largest ESS can are observed for $\beta _2$, $\tau _1$ and $\tau _2$ which indicates that these parameters have the lowest posterior autocorrelation and most efficient exploration of the sample space. The specimen E4 appears to have the lowest ESS for most parameters even after thinning by 1000. This reflects a very high autocorrelation and potentially a substantial uncertainty in the estimation of $\gamma$.

**Table 2:** Effective sample sizes for the MCMC traces of the conditional marginal posterior distributions for E1–E4.

Specimen name	$\alpha$	$\beta _1$	$\beta _2$	$\tau _1$	$\tau _2$	$\gamma$
E1 (thinned by 100)	38544.43	36468.90	40761.65	40000.00	41809.44	39205.76
E2 (thinned by 100)	2832.99	2384.14	22050.89	4243.23	40000.00	2280.00
E3 (thinned by 100)	16694.35	6333.24	21968.43	22730.06	31540.39	13439.44
E4 (thinned by 1000)	1562.40	1504.76	34670.76	2920.24	1941.36	1306.28

Figure 3 illustrates the density plots of parameter posterior distributions. In regards to the model parameters other than $\gamma$, the specimens E1 and E3 have unimodal posterior distributions for most of the parameters. Conversely, E2 and E4 illustrate posterior multimodality for most parameters although their global posterior modes are still well-defined. The posterior distributions for $\tau _1$ are extremely heavy-tailed, their separate density plots are in Supplementary Information Figure S7. Despite having the smallest posterior mean, E3 has the longest tail of $\tau _1$ posterior in relation to other specimens perhaps indicating that the presence of noisy measurements led to the acceptance of extreme proposed $\tau _1$ values. The specimen E4 has a multimodal distribution of $\tau _1$ with the global mode of ca. $6\times 10^{4}$ which is similar to E1 and E2. The posterior variance is much smaller than that of the prior for $\alpha ,\beta _1$ and $\beta _2$ and is somewhat smaller for $\tau _1$ and $\tau _2$ across all of the specimens.

The specimen split between posterior uni-/bimodality of the regression parameters is likely to be related to the accepted values of $\gamma$. Plot (f) illustrates that E1 and E3 have very strongly-defined posterior modes of $\gamma$ with smaller local modes around the lower end of the concentration range. The posterior $\gamma$ density of E2 has a strong global mode, however the mode around 50 mg/L also has a high proportion of posterior density. Similarly, the posterior $\gamma$ density of E4 has a global mode around 45 mg/L with strong peaks around 120 and 150 mg/L. The latter phenomenon can be attributed to the compartmentalisation of the posterior density around the specimen data which results in one/several regions where an intersection of two regressions is likely. This gives rise in bimodality of the posterior distributions of the remaining regression parameters. To illustrate this, Fig. 4 shows the sampled posterior log-density, $\log \pi (\theta |y)$, versus the sampled values of $\gamma$. For E1 and E3 $\log \pi (\theta |y)$ peaks around the posterior mean of $\gamma$. For E2, the $\log \pi (\theta |y)$ is near the posterior mean, however for E4 it does not correspond to a global peak but rather is a ‘weighted average’ of the local peaks corresponding to density peaks in Fig. 3(f). It might be advantageous to retake the E4 specimen measurement in order to obtain a more conclusive estimate of $\gamma$, which would be a focus of a follow-up investigation.

## Discussion

Bayesian inference and MCMC sampling of the posterior distribution can be very useful in the conditions of sparse and noisy data. Figure 5 illustrates the posterior estimation of the changepoint $\gamma$ of the four asphaltene specimens, as well as Bayesian credible intervals^61^. It is clear that our measurements have a greater noise that those reported by e.g. Andreatta et al.^39^ and the use of Bayesian inference has allowed us to estimate the $\gamma$ value as well as the uncertainty region for the entire changepoint model. Note that every data point in Fig. 1 is an average over up to 100 measurements over time. The variation of the ultrasound speed across these measurements is small relative to the overall trend. In our previous study^34^ we have illustrated using pure surfactant solutions that the Resoscan instrument can detect molecular self-association at a similar scale to the asphaltenes. Thus, we infer that the fluctuations in the ultrasound velocity versus concentration for asphaltenes are mainly caused by the physico-chemical properties of the sample.

The changepoint $\gamma$ posterior mean values for E2 and E3 are close to the prior means of 100 mg/L. In contrast, E1 and E4 have posterior means at 78 and 73 mg/L respectively. Also, E4 has the widest 95% confidence interval for $\gamma$ which illustrates the impact of $\log \pi (\theta |y)$ multimodality (Fig. 4). The shaded confidence regions reflect the estimation of the posterior mean of the regression parameters $\alpha , \beta _1$ and $\beta _2$. For E3, the velocity range spans ca. 0.5 m/s which is the largest interval of all the specimens, reflecting the largest posterior variance (Fig. 3a–c). The obtained confidence intervals are consistent with the previous literature findings^20^. For all of the specimens, one point or less lie outside the credible intervals which is consistent with a 5% outlier rate.

Noteworthy, the behaviour of E1 is different to the remaining samples in that the velocity gradient is increasing in the monomeric region as opposed to vice versa in the remaining samples. The gradients in Fig. 5 are controlled by a complex relation between apparent molar quantities, including compressibility and volume (Eq. 3). For example, one of the reasons of the ultrasonic gradient on the monomeric region to be negative is for the apparent compressibility of the monomeric asphaltene to be more than twice that of the solvent. Understanding the cause for this phenomenon is beyond the scope of this work and should be addressed in future studies.

The estimated changepoint values are similar to those we reported using non-Bayesian methods^34^. In the latter study, the architecture of the asphaltenes was linked to their aggregation behaviour by considering the abundance of the long side-chains ($\hbox {C}_{\ge 19}$). As steric repulsion is assumed to arise from the *n*-alkane appendages^20^, it is reasonable to suggest that the longer moieties complicate/delay nanoaggregation more than the shorter ones. The relation between the abundance of side-chains and complexity in aggregation has been studied earlier in e.g. Wang et al.^62^. In our earlier study, side-chains have been obtained by mineralising the aromatic cores and releasing the aliphatic appendages using ruthenium ion catalysed oxidation (RICO)^63, 64^. Asphaltene side-chains are released as *n*-alkanoic fatty acid methyl esters which are the dominant products, and are also extremely volatile. Also, the reaction produces a significant amount of *di*-alkanoic fatty acid *di*-methyl esters and other products which can interfere with the main peaks of interest^63, 65^. Therefore, the results of RICO should be used as indicative. The specimens E1–E4 were reported to have 14%, 21%, 18% and 11% of long *n*-alkanoic side-chains respectively.

Comparing to the present study, the the relative abundance of the long side-chains is linearly related to $\gamma$. In particular, E2 has the largest posterior $\gamma$ mean as well as the greatest abundance of the long side-chains, followed by E3, E1 and E4. This in turn would support the steric hindrance argument. Although this relation is reasonable and exciting, we have illustrated the difficulties in estimating the posterior $\gamma$ for E4. Therefore, the link between the changepoint concentration and the abundance of long side-chains should serve as an an indication of a relation and be tested in subsequent studies. Nonetheless, the combined use of the asphaltene structural properties and the probabilistic modelling of their changepoint have the potential to be powerful in oil spill remediation strategies.

## Conclusions

Asphaltene nanoaggregation ultrasonic characterisation data has been studied using Bayesian inference to produce an estimation of the critical nanoaggregation concentration^20, 39^, herein referred to the changepoint $\gamma$. This is a novel application in the field of the asphaltenes and is superior to the frequentist methods as the uncertainty associated with the aggregation concentration, as well as model parameters, can be quantified. The use of Bayesian inference has allowed to incorporate the literature information about asphaltene $\gamma$, thus helping to navigate through the noise in the measurements. The $\gamma$ estimation of the four samples indicated values consistent with literature, although an earlier onset of aggregation has also been suggested linked to a lower abundance of the longer aliphatic appendages. Despite the noise in the data, the Bayesian sampling scheme was able to recover the regression behaviour and estimate the $\gamma$ and it’s confidence intervals for all of the specimens. This illustrates that the combined of prior information and experimental data likelihood is extremely useful in conditions of data sparsity and noise.

Given an appropriate prior distribution specification our model can be applied to any asphaltene characterisation data that is assumed to follow a changepoint regression behaviour and has uncertainty, such as calorimetry^19, 66^ and nuclear magnetic resonance^67^. The computational burden of the proposed sampling scheme is very low and can be run within minutes.

Further developments of this work include a hierarchical Bayesian structure of the single-changepoint model that would allow to estimate the regression coefficients by borrowing strength from all of the specimens/pooling the data. A two-changepoint model may also be proposed to estimate the $\gamma$ whereby the region of aggregation is modelled by an appropriate stochastic process. The latter which would allow a more flexible structure for a process combining monomeric and aggregated behaviours if the complexity of the asphaltene monomers^22^ is too high for the model by Zielinski et al. to be appropriate. Additional geochemical investigations can also complement the current study, for example further understanding the composition and architecture of the aliphatic moieties of the asphaltenes through elemental analysis.

## Supplementary information

Supplementary material 1

## Data Availability

The data sets used in this study are available from Newcastle University Research Data Repository 10.25405/data.ncl.14206862.v1. Computer algorithms and further information can be obtained from the corresponding author on reasonable request.
