# Supplementary material for: Estimating the asphaltene critical nanoaggregation concentration region using ultrasonic measurements and Bayesian inference
Source: Sci Rep. 2021 Mar 23;11:6698. doi: 10.1038/s41598-021-85926-8 (PMC7988144; doi:10.1038/s41598-021-85926-8)
Supplement: Supplementary file 1 — Supplementary material 1 [file 41598_2021_85926_MOESM1_ESM.pdf]

# Estimating the asphaltene critical nanoaggregation concentration region using ultrasonic measurements and Bayesian inference. Supplementary Information.

**Aleksandra Svalova[1,*], David Walshaw[1], Clement Lee[4], Vasily Demyanov[3], Nicholas G Parker[1], Megan J Povey[5], and Geoffrey D Abbott[2]**

[1]School of Mathematics, Statistics and Physics, Newcastle University, Newcastle upon Tyne, NE1 7RU, UK
[2]School of Natural and Environmental Sciences, Newcastle University, Newcastle upon Tyne, NE1 7RU, UK
[3]Energy Academy, Heriot-Watt University, Edinburgh, EH14 4AS, UK
[4]Department of Mathematics and Statistics, Lancaster University, Lancaster, LA1 4YF, UK
[*]alex.svalova@ncl.ac.uk
[5]School of Food Science and Nutrition, University of Leeds, Leeds, LS2 9JT

## ABSTRACT

Bayesian inference and ultrasonic velocity have been used to estimate the self-association concentration of the asphaltenes in toluene using a changepoint regression model. The estimated values agree with the literature information and indicate that a lower abundance of the longer side-chains can cause an earlier onset of asphaltene self-association. Asphaltenes constitute the heaviest and most complicated fraction of crude petroleum and include a surface-active sub-fraction. When present above a critical concentration in pure solvent, asphaltene "monomers" self-associate and form nanoaggregates. Asphaltene nanoaggregates are thought to play a significant role during the remediation of petroleum spills and seeps. When mixed with water, petroleum becomes highly-viscous thus expensive to remove from the water column by conventional methods. The main reason of this difficulty is the presence of highly surface-active components in petroleum, also known as the asphaltenes. The nanoaggregates are thought to surround the water droplets, making the water-in-oil emulsions extremely stable. Due to their molecular complexity, modelling the self-association of the asphaltenes can be a very computationally-intensive task and has mostly been approached by molecular dynamic simulations. Our approach allows the use of literature and experimental data to estimate the nanoaggregation and it's credible intervals. It has a low computational cost and can also be used for other analytical/experimental methods probing a changepoint in the molecular association behaviour.

## Supplementary Information

### Conditional posterior distributions

$$\alpha|\mathbf{x},\theta_{-\alpha} \sim N\left(A_\alpha B_\alpha^{-1}, B_\alpha^{-1}\right), \tag{S1}$$

$$A_\alpha = \tau_1 \sum_{i=1}^{n_1}(y_i - \beta_1 x_i) + \tau_2 \sum_{i=n_1+1}^{n}(y_i - \gamma(\beta_1-\beta_2) - \beta_2 x_i) + \frac{a}{s_\alpha^2}, \quad B_\alpha = n_1\tau_1 + n_2\tau_2 + s_\alpha^{-2}.$$

$$\beta_1|\mathbf{x},\theta_{-\beta_1} \sim N\left(A_{\beta_1} B_{\beta_1}^{-1}, B_{\beta_1}^{-1}\right), \tag{S2}$$

$$A_{\beta_1} = \tau_1 \sum_{i=1}^{n_1} x_i(y_i-\alpha) + \tau_2\gamma \sum_{i=n_1+1}^{n}(y_i - \alpha - \beta_2(x_i-\gamma)) + \frac{b_1}{s_{\beta_1}^2}, \quad B_{\beta_1} = \tau_1 \sum_{i=1}^{n_1} x_i^2 + \tau_2 n_2 \gamma^2 + s_{\beta_1}^{-2}.$$

$$\beta_2|\mathbf{x},\theta_{-\beta_2} \sim N\left(A_{\beta_2} B_{\beta_2}^{-1}, B_{\beta_2}^{-1}\right), \tag{S3}$$

$$A_{\beta_2} = \tau_2 \sum_{i=n_1+1}^{n}(x_i-\gamma)(y_i-\alpha-\gamma\beta_1) + \frac{b_2}{s_{\beta_2}^2}, \quad B_{\beta_2} = \tau_2 \sum_{i=n_1+1}^{n}(x_i-\gamma)^2 + s_{\beta_2}^{-2}.$$

$$\tau_1|\mathbf{x},\theta_{-\tau_1} \sim Ga\left(0.5n_1+\rho_1, 0.5\sum_{i=1}^{n_1}(y_i-\alpha-\beta_1 x_i)^2+\phi_1\right). \tag{S4}$$

$$\tau_2|\mathbf{x},\theta_{-\tau_2} \sim Ga\left(0.5n_2+\rho_2, 0.5\sum_{i=n_1+1}^{n}(y_i-\alpha-\gamma(\beta_1-\beta_2)-\beta_2 x_i)^2+\phi_2\right). \tag{S5}$$

### Synthetic data study

We tested our proposed MCMC sampling scheme on two synthetic data sets, to match our data[1] and that published by Andreatta, *et al.*[2]. In particular, we have created a synthetic E2 data set using parameter values that are close to the E2 posterior parameter estimates and a synthetic data set to equal the ultrasonic characterisation of UG8 asphaltene[2]. The parameter values for the synthetic data sets are listed in Table S1 and the data sets are illustrated in Figure S1. Noteworthy, the precision of the synthetic UG8 data set is two orders of magnitude higher than that of the synthetic E2.

The MCMC sampler was run with the set-up and prior distributions consistent with those described in the Methodology section. The resulting MCMC traces are illustrated in Figure S2 where good mixing can be observed for all posterior distributions. As expected, the mixing for the synthetic UG8 data is more efficient than that for E2 due to the lower noise in the data. For E2, the sampler is accepting more extreme values for the conditional posterior distributions of $\beta_1$ and $\tau_1$ than for the remaining parameters. Nevertheless, the convergence is apparent for all parameters.

Figure S3 illustrates the posterior densities for the two synthetic data sets. Consistent with the earlier trace plot, the posterior distributions are smooth and have a clearly-defined mode. The E2 posterior distributions of $\beta_1$ and $\tau_1$ have heavier tails, again, as indicated by their trace plots. All of the posterior means are close to the true parameter values, except for the synthetic UG8 $\tau_1$ and $\tau_2$. This could be explained by the slight mis-estimation of the remaining regression parameters which change the estimation of the posterior precision in both monomeric and aggregated regions. The values of the parameter prior means do not seem to have too strong of an influence on detecting true parameter values, as for example, for the synthetic UG8 $\alpha$ and $\beta_2$ the prior means are out of the 95% density region. Most importantly, the posterior estimation of the changepoint $\gamma$ is consistent with their true values for both data sets. For the synthetic E2 $\gamma$, slight multimodality may be observed which we attribute to the compartmentalisation of the density around data points. Additionally, the posterior mean of $\gamma$ is overestimating the true value by ca. 14 mg/L. Although this is unfortunate, the global posterior mode is very close to the true $\gamma$, thus this may also be consulted in application. Noteworthy, the $\gamma$ posterior distribution for the synthetic E2 data set also has a local mode around 40 mg/L. This can be expected in noisy data sets and should be interpreted with caution.

Additionally, Figure S4 illustrates the distribution of the posterior log-density against the sampled $\gamma$ values. Clearly, for both data sets the posterior means and true parameter values correspond to the peak in posterior log-density. This indicates that the most likely set of model parameters given the model, prior distributions and the synthetic data corresponds to the posterior mean estimates of $\gamma$.

Figure S5 illustrates the posterior predictive distributions of the changepoint model for the synthetic UG8 and E2 data sets. The posterior predictive intervals (shaded in cyan) capture all of the data points, except an outlier for E2 around 300 mg/L which is consistent with the normality assumption of a 5% error rate. The mean model estimation matches the actual regressions very well. For the synthetic UG8 data, the posterior $\gamma$ estimation is very close to the true value of 164 mg/L. For the synthetic E2 data, the posterior mean of $\gamma$ is overestimating the true value of $\gamma$ by ca. 14 mg/L, nevertheless the predicted model is very close to the true values.

Therefore, we have illustrated that our one-changepoint model and MCMC scheme can produce reliable estimates even in noisy conditions.

## References


1. Svalova, A., Parker, N. G., Povey, M. J. W. & Abbott, G. D. Determination of asphaltene critical nanoaggregate concentration region using ultrasound velocity measurements. *Sci. Reports* **7**, 1–11 (2017).
2. Andreatta, G., Bostrom, N. & Mullins, O. C. High-Q ultrasonic determination of the critical nanoaggregate concentration of asphaltenes and the critical micelle concentration of standard surfactants. *Langmuir* **21**, 2728–2736 (2005).

| Synthetic specimen name | $\alpha$ | $\beta_1$ | $\beta_2$ | $\tau_1$ | $\tau_2$ | $\gamma$ |
|---|---|---|---|---|---|---|
| E2 synthetic | 1304.585 | $-5.5\times10^{-4}$ | $5\times10^{-5}$ | 4127.97 | 2384.56 | 107.658 |
| UG8 synthetic | 1307.099 | $-2\times10^{-5}$ | $6\times10^{-5}$ | 400000 | 150000 | 164 |

**Table S1.** Parameter values (to three places) used in the creation of synthetic data sets. The underlying model is consistent with the formalism (M1).

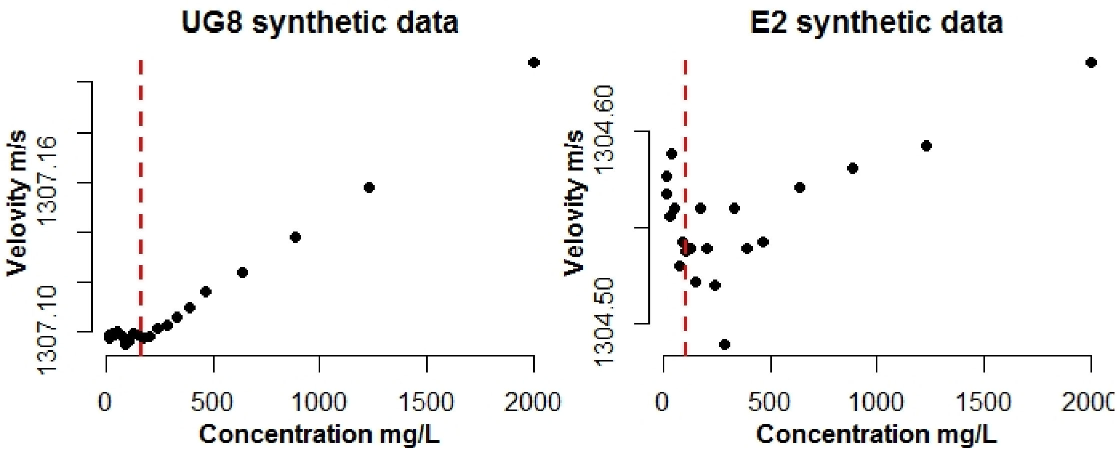


**Figure S1.** Plots of synthetic data sets E2 and UG8. True $\gamma$ values are indicated by the red dashed lines.

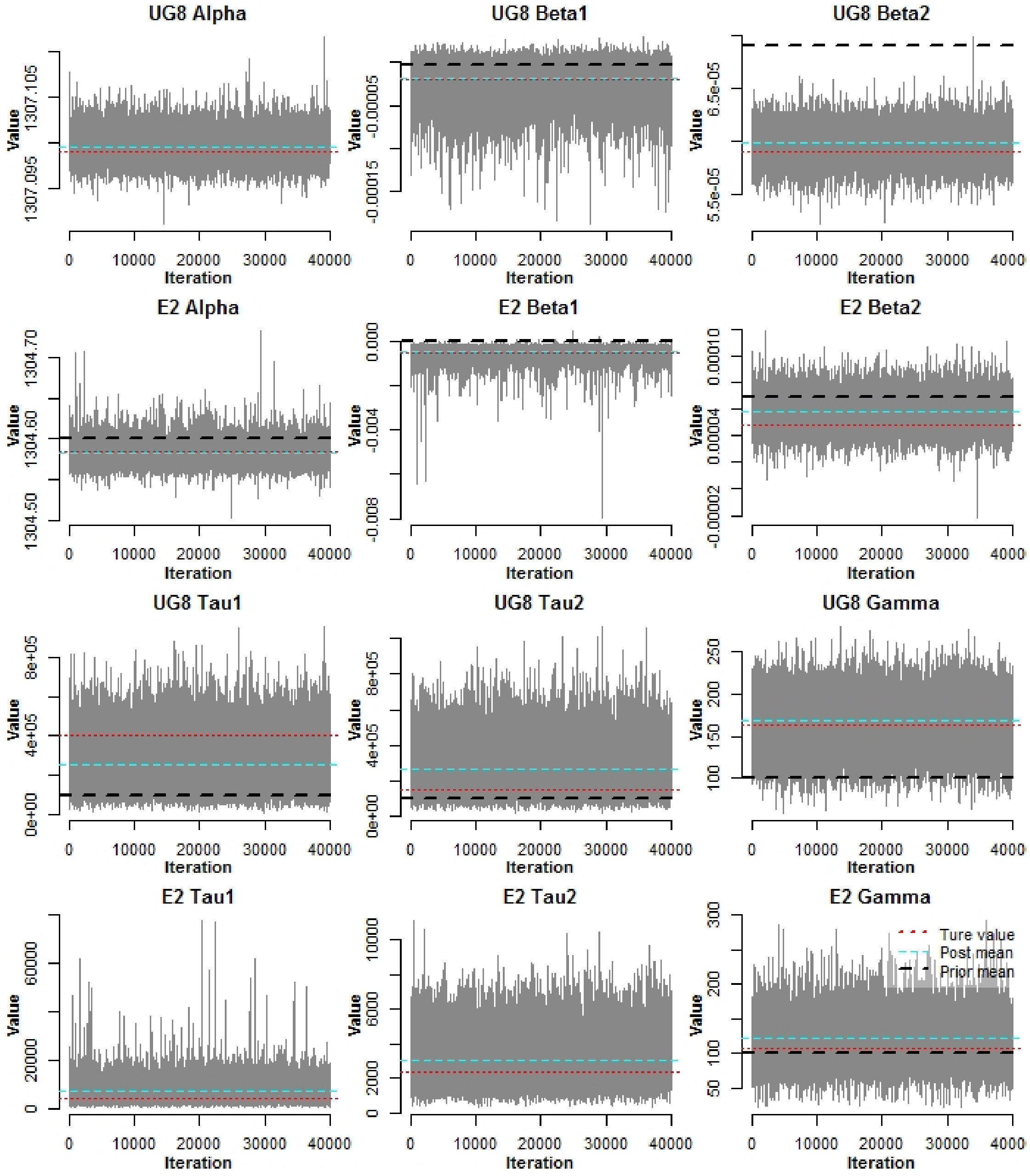


**Figure S2.** Trace plots of the MCMC draws of the posterior distributions of the single changepoint model parameters using synthetic data sets. Red lines indicate true parameter values, cyan lines indicate posterior means, black lines indicate prior means.

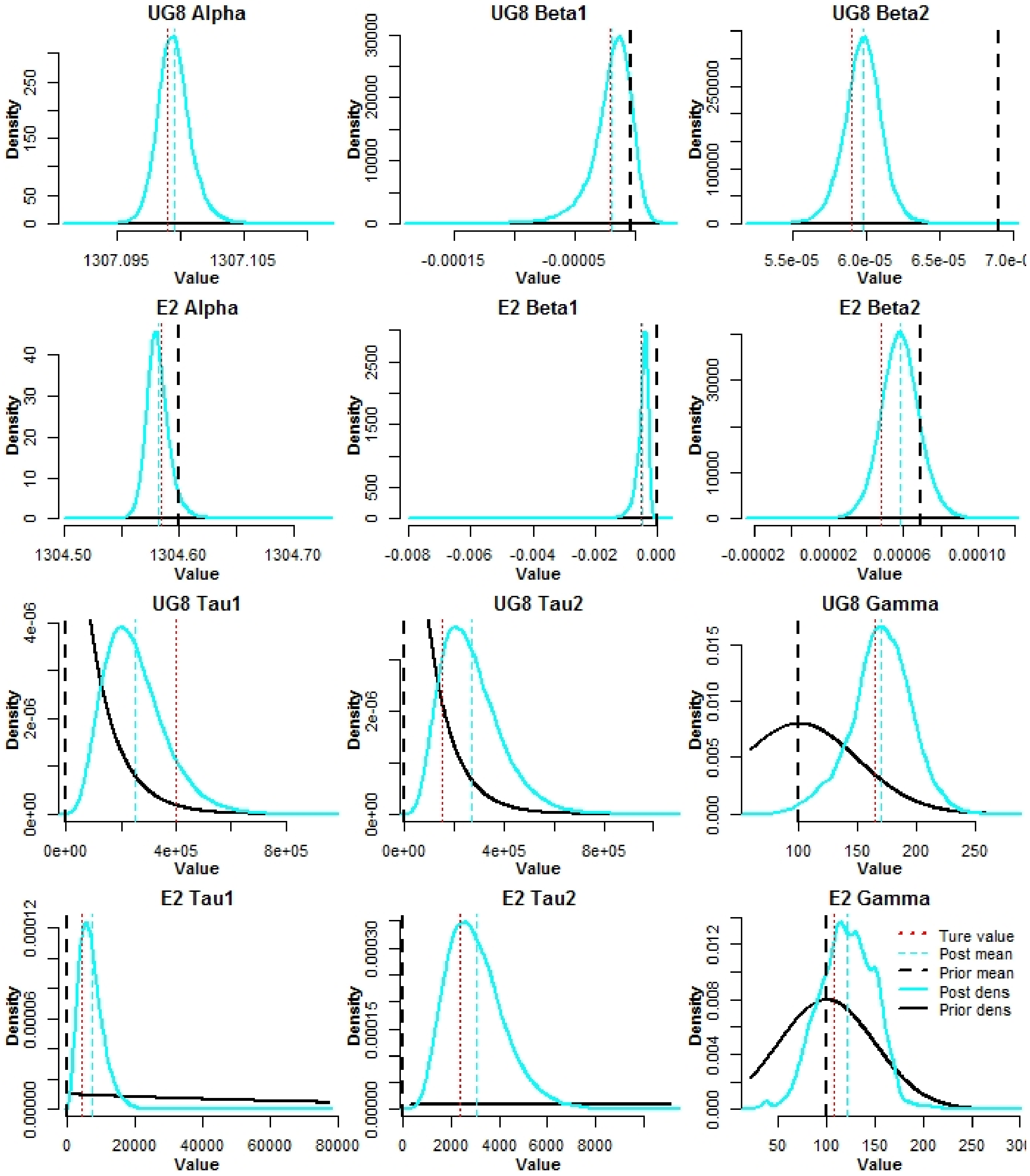


**Figure S3.** Density plots of the posterior distributions of the single changepoint model parameters for synthetic data sets.

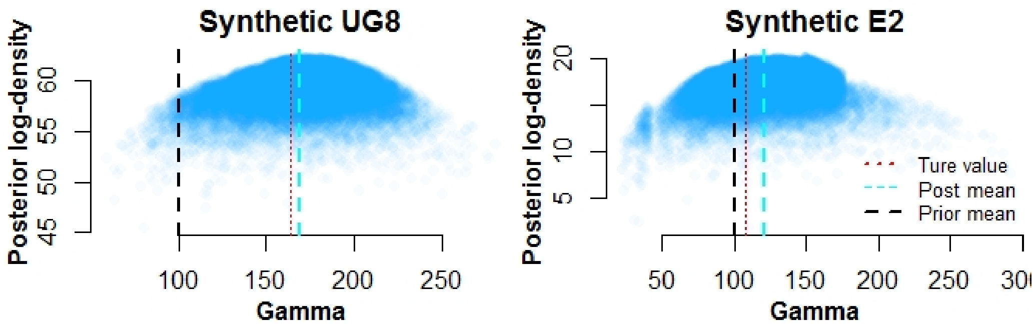


**Figure S4.** Gamma values sampled during the Metropolis step of MCMC sampling versus the posterior log-density.

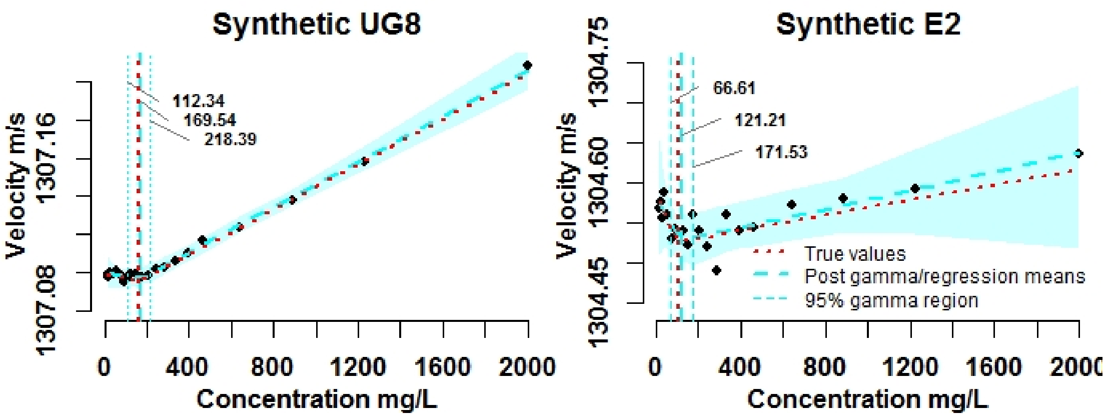


**Figure S5**. Posterior predictive distributions of the synthetic UG8 and E2 samples.

## Additional plots

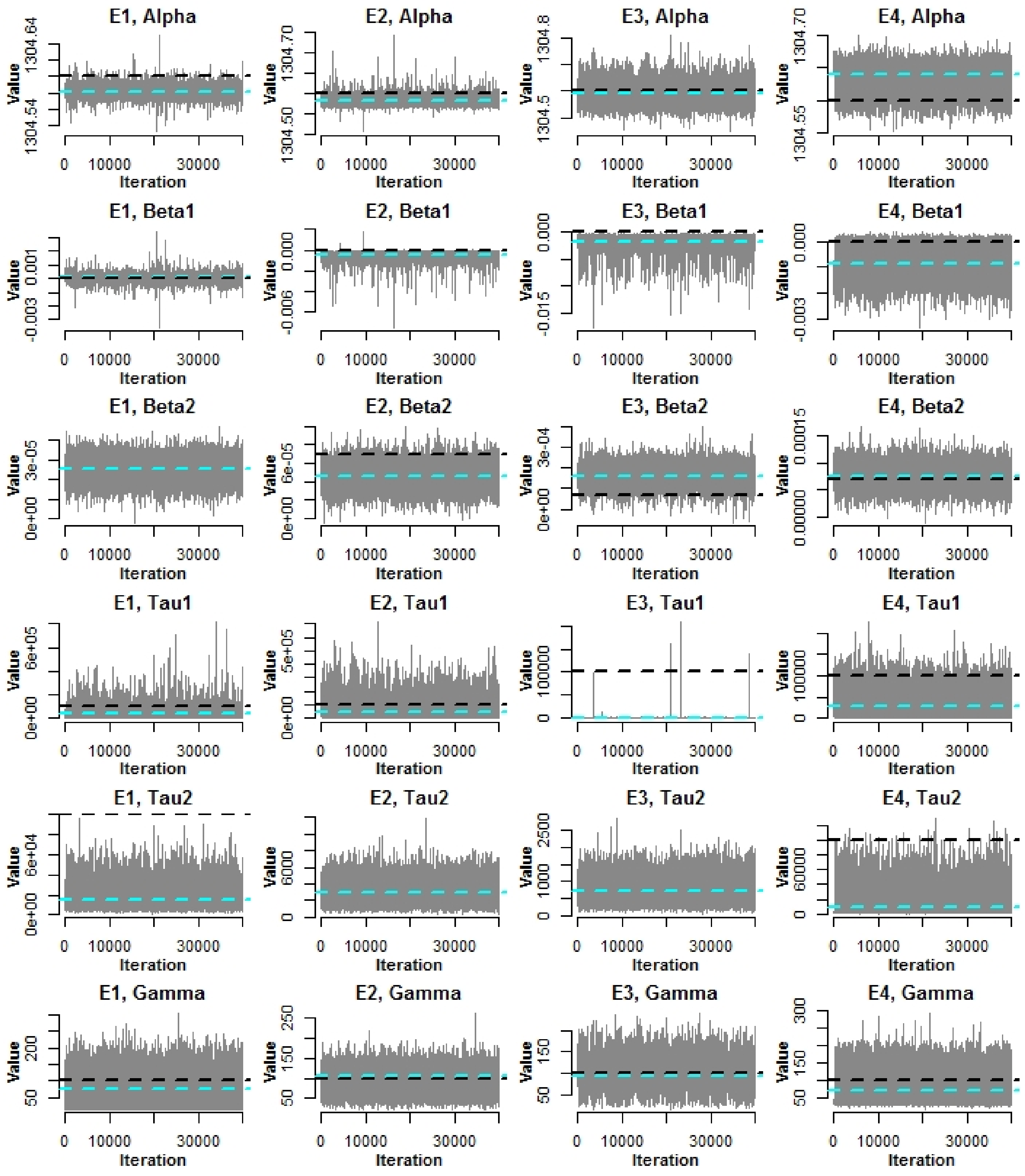


**Figure S6.** Trace plots of the MCMC draws of the posterior distributions of the single changepoint model parameters. Blue lines indicate prior means and red lines indicate posterior means.

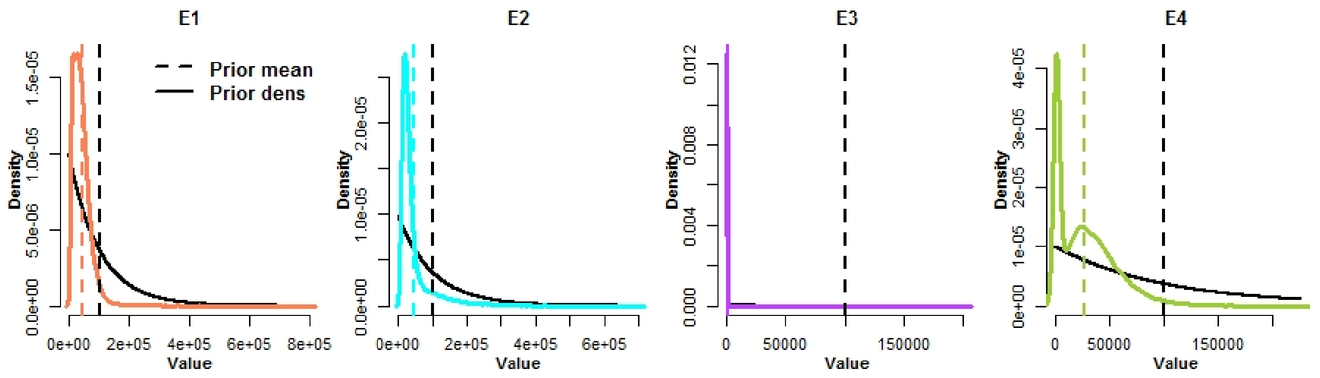


**Figure S7.** Density plots of the posterior distributions of $\tau_1$.
